# Medication safety research by observational study design

**DOI:** 10.1007/s11096-016-0285-6

**Published:** 2016-03-22

**Authors:** Kim S. J. Lao, Celine S. L. Chui, Kenneth K. C. Man, Wallis C. Y. Lau, Esther W. Chan, Ian C. K. Wong

**Affiliations:** Department of Pharmacology and Pharmacy, Centre for Safe Medication Practice and Research, Li Ka Shing Faculty of Medicine, The University of Hong Kong, 2/F Laboratory Block, 21 Sassoon Road, Pok Fu Lam, Hong Kong SAR China; School of Pharmacy, University College London, 29-39 Brunswick Square, London, WC1N 1AX UK

**Keywords:** Methodology, Observational study, Pharmacoepidemiology

## Abstract

Observational studies have been recognised to be essential for investigating the safety profile of medications. Numerous observational studies have been conducted on the platform of large population databases, which provide adequate sample size and follow-up length to detect infrequent and/or delayed clinical outcomes. Cohort and case–control are well-accepted traditional methodologies for hypothesis testing, while within-individual study designs are developing and evolving, addressing previous known methodological limitations to reduce confounding and bias. Respective examples of observational studies of different study designs using medical databases are shown. Methodology characteristics, study assumptions, strengths and weaknesses of each method are discussed in this review.

## Impacts on practice

- The advancement of observational study design allows pharmacists and researchers to gain better understanding of the incidences and causes of adverse drug events. This can enhance the patient safety and ultimately better care for patients.

## Introduction

The importance of observational studies in the evaluation of drug safety has been recognised in recent decades, along with the ongoing interest about drug adverse events over time. Data generated from observational studies supplement premarketing experimental trials, especially in situations where the outcome of drug exposure is rare, delayed or observed in specific subgroups. In such cases, large databases offer a platform with relatively large sample size, long follow-up period and few ethical issues, which are cost-effective and efficient compared to interventional studies.

Since the 1980’s, substantial observational studies have been conducted using large databases. Databases used should ideally include a large and stable population, be representative and verifiable. Based on the source and type of data, databases are generally divided into two types, administrative databases and clinical databases. Administrative databases, include the Medicaid (United States [US]) and the National Health Insurance Research Database (NHIRD, Taiwan), which functions primarily as insurance claims databases [1, 2]. Clinical databases, on the other hand contain electronic medical records entered for clinical use and patient monitoring. Such databases include the Clinical Data Analysis and Reporting System database (CDARS, Hong Kong) and the Clinical Practice Research Datalink (CPRD, United Kingdom [UK]) [1, 2]. With both types of database, observational studies can be conducted using different study designs.

Considered as fundamental or traditional methods, cohort and case–control design are widely applied but can be vulnerable to confounder and selection bias. To tackle methodological limitations, new study designs for observational studies have been developed to eliminate or at least minimise the effect of time-invariable factors. Within-individual designs, including self-controlled case series study (SCCS) [3] and case-crossover study (CCO) [4], introduced since the early 1990’s, are now widely used for observation and evaluation of drug safety and effectiveness.

The aim of this review is to introduce readers to the design of conventional and innovational observational methods for drug safety and effectiveness research. This review will present the characteristics, assumptions, strengths and limitations of each method. Examples of each method are also given to illustrate their application.

## Cohort study

### Characteristics

**Fig. 1:**
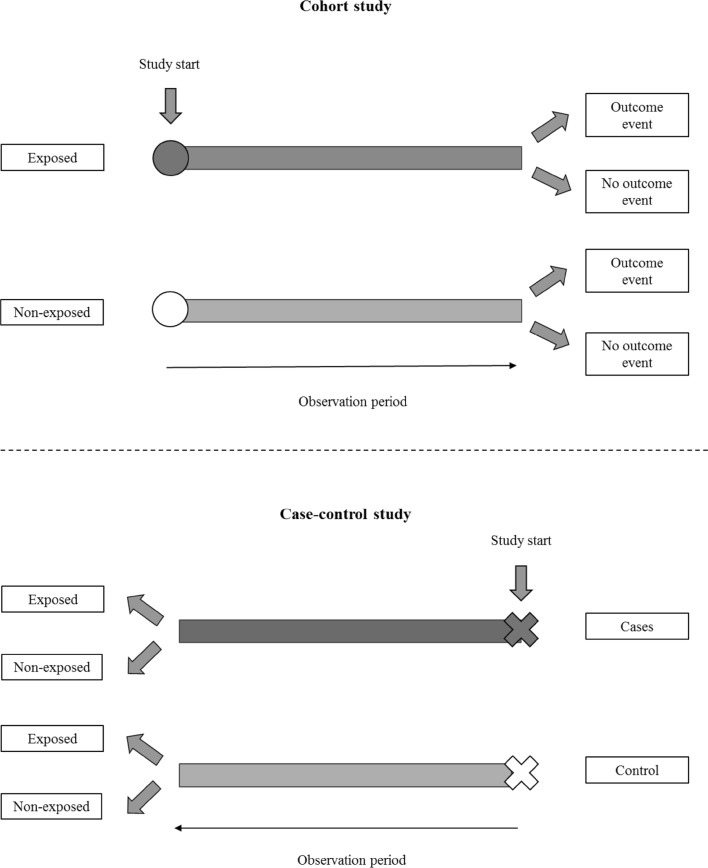
Cohort and case control study designs. In a cohort study, subjects are classified as an exposed or non-exposed group based on their drug exposure status at study commencement. Subjects are then followed up over time to identify any occurrence of the outcome of interest. In a case–control study, subjects are classified as case and control (non-case) at study commencement. Subjects with the outcome of interest are defined as cases, while subjects without the outcome of interest are defined as controls. Information is collected retrospectively to identify any previous drug exposure

A cohort study is used to examine causal factors [5]. This type of study recruits a group of subjects to represent the population of interest. Subjects are included at commencement of the study and classified as exposed (treatment group) or non-exposed (control group), based on their drug exposure status (Fig. 1). In some cohort studies, the control group could have other treatment(s) [6] or a different dose of the same treatment [7]. Subjects are then followed up over time to identify the incidence of outcome of interest, usually adverse events in the treatment and control groups.

Cohort studies can either be prospective or retrospective. Prospective cohort studies are carried out from present time to future. It usually starts with the collection of specific exposure data, but there may be a long wait for events to occur, particularly where the outcome of interest is a chronic event. Studies can therefore be expensive to carry out and are prone to high dropout rates. Conversely, retrospective cohort studies look at outcome of interest from a time-point in the past up to the intended study period. The advantage of retrospective cohort studies is that the information is available immediately. However, there may be difficulty in tracing subjects and further information required relies solely on the already recorded data for such studies. Furthermore, the validity of the database should be carefully considered, as most databases applied currently may not have been established for research purposes.

### Strength and weakness

Advantages of cohort studies include the fact that exposure status is determined before the outcome of interest, which is less likely to be prone to bias. Further, multiple outcomes can be explored at a time. Also, due to the nature of recruitment, cohort studies are suitable for studying rare exposures. On the other hand, prospective cohorts usually take a long time and are therefore costly. A very large sample size is also required for rare outcomes.

### Example

The use of dabigatran, an inhibitor of thrombin, increases risk of gastrointestinal bleeding (GIB). However, it is not clear whether gastroprotective agents (GPAs) prevent GIB in dabigatran users [8]. Using a retrospective cohort study design, Chan et al. [8] investigated the association between the use of GPAs and the risk of GIB in dabigatran users. Utilising electronic medical records from CDARS, Chan et al. identified a group of dabigatran users (the cohort) between 2010 and 2013. Among the cohort, patients who had a prescription of either histamine type-2 receptor antagonists and/or proton pump inhibitors during follow-up were defined as exposed to GPAs whilst others were defined as unexposed (control). Included patients were then followed up until the end of the observation period to ascertain whether they had a diagnosis of GIB (outcome). Retrospective cohort studies require accurate records for the exposures and outcomes, Chan et al. used dispensed medications as exposures and verified diagnosis records as outcomes to enhance validity as misclassification of exposures and/or outcomes will bias the results.

To compare the risk of GIB between GPA users and non-users, Poisson regression was used to determine the incidence rate ratio (IRR) with 95 % confidence intervals (CI), among patients who were taking dabigatran. Adjustment in the regression model was made to control for baseline medical conditions and use of concurrent medications. The study showed that a reduction of 48 % in the risk of GIB was found in GPA users as compared with nonusers (IRR 0.52; 95 % CI 0.35–0.77).

## Case–control study

### Characteristics

As in cohort studies, the purpose of case–control studies is to evaluate the association between risk factors and outcome of interest [5]. In contrast to cohort studies, however, individuals in the population with the outcome of interest are identified at the onset (Fig. 1). Risk factors or exposure information is collected retrospectively. Individuals with the outcome are determined as cases. Individuals who do not have the outcome of interest, the controls, are also included in the study. The case–control study design is often used in the study of rare outcomes or as a preliminary study where little is known about the association between the risk factor and disease of interest.

Case definition should be precise to distinguish between stages, severity or subtypes of disease and to define a measure of health status so that cases and controls for the study can be chosen appropriately [9]. Controls should come from the same population at risk of disease, should not have the disease and should be representative of the target population. The selection of suitable controls requires great care in the prevention of bias. Cases and controls can be matched to address confounding factors which might contribute to the development of disease and confound the causal association under investigation. A case–control study conducted in a specified cohort is called a nested case–control study [10]. For many research questions, the nested case–control design offers a cost effective option and reduce the time required for data collection and analysis compared with the full cohort approach, with relatively minor loss in statistical efficiency.

### Strength and weakness

Case–control studies are relatively quick to run and incur lower costs compared to cohort studies. Case–control studies are particularly suited to the study of rare diseases as the diseased are selected at the outset of the study. The disadvantages, however, include difficulties in selecting proper cases and controls. Also, it is not possible, to calculate the incidence of the disease in case–control studies. However, incidence can be estimated in the nested case–control study.

### Example

Combined oral contraceptives are effective in preventing pregnancy in general. They have, however, measurable side effects such as venous thromboembolism (VTE), which is a potentially fatal but avoidable prolonged event [11]. A study by Vinogradova et al. [11] investigated the association between the use of combined oral contraceptives and risk of VTE with two clinical databases (CPRD and QResearch) in UK. Study population includes all women without records of VTE before the study, aged 15–49 years, who were registered with the study practices between 2001 and 2013. For both databases, they matched each case with up to five controls by birth year and from the same general practice. Each control was allocated an index date, which was the date of first VTE diagnosis for the matched case. Exposure to hormonal contraceptive drugs was based on prescription information in the last year before the index date.

To prevent heterogeneity between databases, two nested case–control studies within each dataset and separate analyses were conducted. Conditional logistic regression model was applied to obtain odds ratios (ORs) with 95 % CI. In addition, imputation model was applied in handling missing data for body mass index, smoking status, and alcohol consumption [11]. In total, they identified 7334 incident VTE cases from CPRD and 8211 cases from QResearch within the study period. Crude incidence of VTE cases per 10,000 women years was 5.9 in CPRD and 6.1 in QResearch. For the analyses combining results from both databases, current use of any combined oral contraceptive was associated with a significantly increased VTE risk (adjusted OR 2.97, 95 % CI 2.78–3.17) compared with no exposure in the last year.

Cohort and case–control designs are fundamental methods in observational pharmacoepidemiological research and have been widely applied. However, as discussed above, validity of the results can be affected by biases and confounding effects [12].

## Within-individual design

To reduce confounding by using each case as their own control and eliminate between-individual confounding by time-invariant factors, within-individual designs are proposed. Such time-invariant factors include socioeconomic status, family and personal medical history and genetic factors, which would otherwise be difficult to adjust for using statistical methods.

A within-individual design, also called case-only or self-controlled design, is a modified version of the traditional epidemiological methodologies where there is comparison between different observation periods within the same person to estimate an odd ratio or rate ratio [13–15]. Two main types of within-individual designs are commonly used in epidemiological research: the self-controlled case series and case-crossover study. Both designs compare observation periods within the same individual thus only subjects with the outcome of interest are identified.

## Self-controlled case series (SCCS)

### Characteristics

**Fig. 2:**
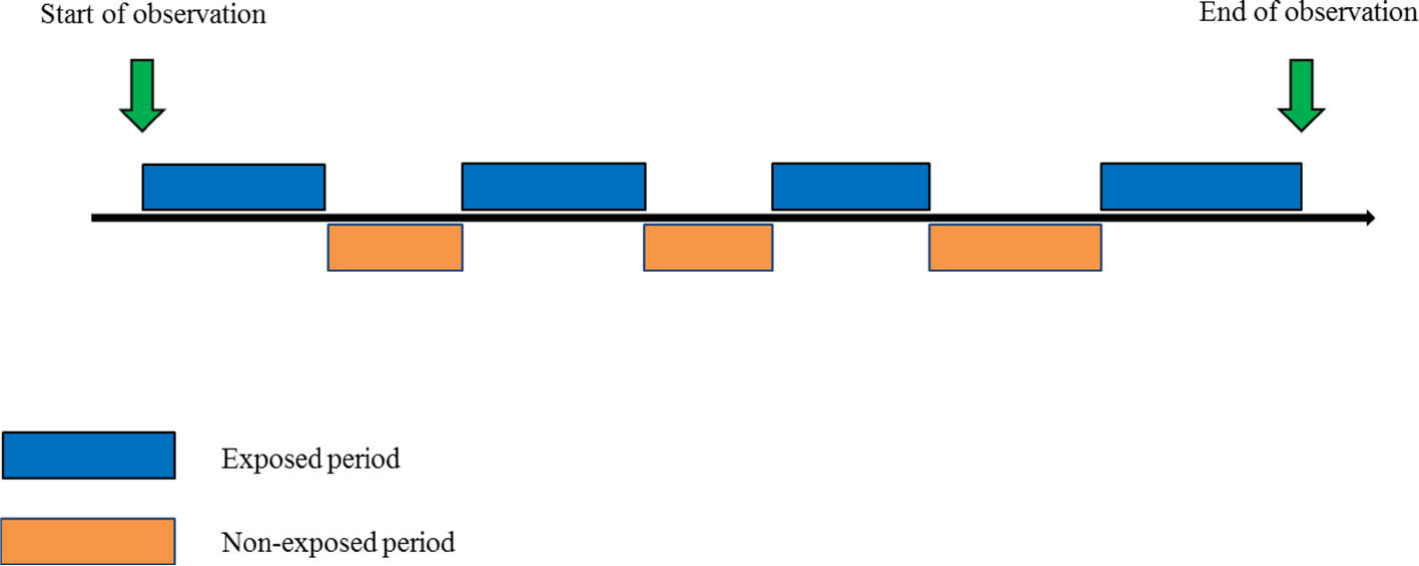
Self-controlled case series study design. Only cases are included in a self-controlled case series study. For each case, within a pre-defined observation period, the time period exposed to the drug is defined as exposed period, while the time period not exposed to the drug is defined as non-exposed period. The rate of the outcome event during the exposed periods is compared with that during the non-exposed periods

The SCCS was first described by Farrington [3] in the application of vaccine-associated mumps meningitis. It was developed to investigate the association between adverse reactions subsequent to vaccination and is now a commonly used study design in pharmacoepidemiological studies [15–18]. Using this method, a relative incidence is derived by comparing the rate of events during the exposed period with the rate during non-exposed periods (Fig. 2). The exposed period is regarded as fixed, whilst the occurrence of events is random [14].

### Assumptions

Three assumptions should not be violated when applying SCCS design [19]. First, events should be rare or independent of each other. For non-recurrent events (such as incident events), the risk over the study period should be small and recurrent events should be independent of each other since we assume the events occur at random in this study design.

Secondly, the occurrence of the event should be independent of the exposures. The occurrence of the event will affect the probability of subsequent exposure and bias the estimate when this assumption is violated, for example, the event is an indication or contraindication of the exposure.

Lastly, the occurrence of the event or any subsequent conditions stemming from the event should not censor the observation period. A typical example of an event that will censor the observation period is death.

### Extension

Several extensions of the SCCS were developed in the past decade to account for the bias induced when the event censors the subsequent observation period [20–22], and can be applied with specific conditions.

### Strength and weakness

The major strength of SCCS is that time-invariant confounding factors (both measured and unmeasured) are inherently controlled in the model because within person comparisons are made. Temporal variables such as age and progression of disease can also be accounted by subdividing the observation period of each subject into calendar years or age categories. In addition, SCCS is less data-intensive compared to classical cohort and case–control study designs since only cases are sampled.

Application to certain outcomes of interest or exposure could be limited by assumptions. However, modified versions of the design were developed to minimise bias. This design is also limited to single outcomes of interest. It also does not provide estimates of absolute incidence but only relative incidence.

### Examples

Several studies are selected to be discussed in detail on how this design was applied and what was done to abide to the assumptions of SCCS. Chui et al. [15] investigated the association between the use of oral fluoroquinolones and the development of retinal detachment using two databases from Hong Kong and Taiwan (CDARS and NHIRD). In this study, the outcome of interest was retinal detachment, where the first event will affect the subsequent re-occurrence of events. In such cases, the incident event of each subject was considered only in the analysis so that the assumption that events should be independent of each other was not violated.

As mentioned previously, the occurrence of the event should not affect the probability of exposure. Douglas et al. [17] conducted a SCCS to investigate the use of orlistat and acute liver injury. They removed the period prior to orlistat exposure from the non-exposed period to assess whether orlistat is temporarily affected by the event. Douglas et al. demonstrated an increased risk of acute liver injury in both pre-exposure and during exposure of orlistat which suggested a non-causal relationship.

Brauer et al. [16] investigated the use of antipsychotics and the risk of myocardial infarction (MI). Since MI may increase the short-term risk of death, the use of SCCS may result in bias. Therefore, they applied the extended SCCS method, which removes this assumption by re-parameterising the SCCS model. They found a significant association between antipsychotics and the risk of MI with an additional validation of a case–control study.

SCCS is also applicable for evaluating the effectiveness of medication in practice. Man et al. [23] used SCCS to evaluate the effectiveness of methylphenidate in the reduction of Accident and Emergency (A&E) admission in children with attention deficient hyperactivity disorder due to trauma. 10 % reduction of A&E admission due to trauma during treatment period was shown in this study.

## Case-crossover design (CCO)

### Characteristics

**Fig. 3:**
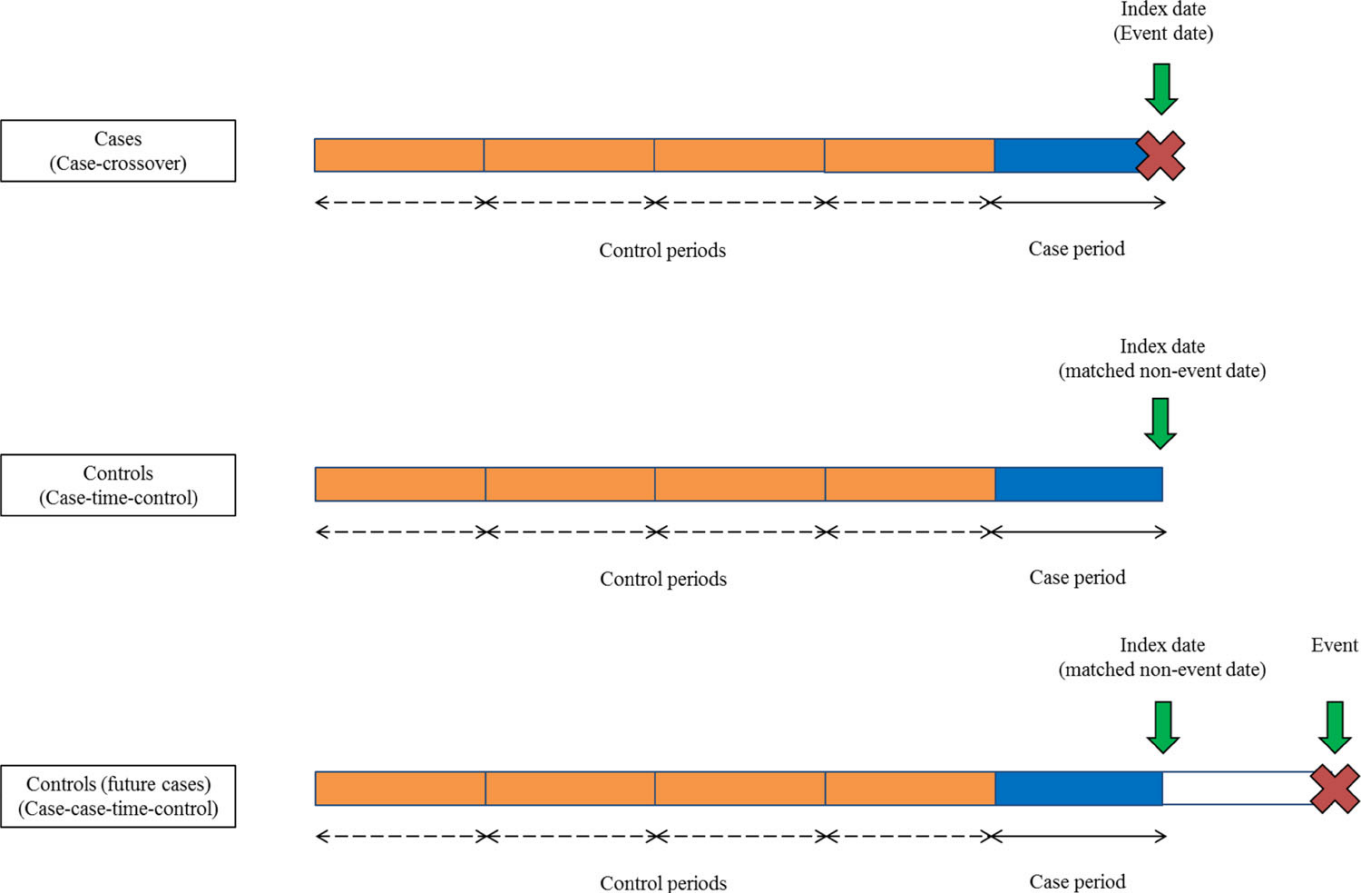
Case-crossover, case–time–control, and case–case–time–control study designs. In a case-crossover study, each case acts as a self-control from previous experience. Case period is defined as the time just before the occurrence of outcome event, while the control period is defined as the time preceding the case period. The drug exposure status during the case period is compared to that during the control period. In a case–time–control study, non-cases are sampled as controls to estimate the effect of exposure time-trend among the cases. Case–case–time–control study is an extension of a case–time–control study, where controls are sampled from future cases instead of non-cases

The CCO, another within-individual design, was developed by Maclure [4] to investigate the risk of acute events. For each case, the time just before an outcome event is defined as case-period, and the preceding times are defined as control-periods (Fig. 3). The exposure status during case-period is compared to that in the control-periods, typically using ORs.

### Assumptions

The CCO design is one of the most efficient study designs for investigating the association of transient exposures with acute outcomes [4, 24]. Although CCO and SCCS depend on within-individual comparison, the requirement for event-independent observation period censoring in SCCS does not apply to the CCO design because the observation period after the event occurrence is not considered in the analysis. The CCO design also assumes the exposure has a stable trend over time. Indeed, several methods have been developed to address the bias of CCO when exposure-time trend exists [25, 26].

### Extension

#### Case–time–control design (CTC)

The CTC design [25] combines CCO with the case–control design. CTC assumes the ORs obtained from the CCO analysis is the combined effects of time-trend and exposure [13]. To obtain the effect of exposure, results will be adjusted for bias from the effect of time-trend, which is estimated using concurrent at-risk time exposure status of non-cases (Fig. 3). In this way, CTC does not require a stable exposure-time trend.

#### Case–case–time–control (CCTC)

The CCTC design [13] is an extension of CTC where controls are sampled from future cases instead of non-cases (Fig. 3). CCTC is suggested to be less prone to protopathic bias [27], which occurs when early symptoms of an outcome leads to exposure. Indeed, it has been demonstrated that future cases can provide a better estimate of the exposure-time trend among the cases compared to non-cases [13].

### Strength and weakness

A major advantage of CCO is that it eliminates confounding effects fixed over time within the individual. However, CCO is subject to bias from confounders that vary with time [25, 26]. Although CTC and CCTC accounts for this potential bias, they may reintroduce control-selection bias if the external controls are not well-matched [28]. Therefore, depending on the magnitude of exposure-time trends and the suitability of the matched-control group, the performance of CCO, CTC, and CTCC could vary in different scenarios.

### Example

Unlike SCCS, the use of CCO design is not prone to the assumption of event-dependent censoring of observation period. Using CCO as an internal validation of the SCCS analysis, Wong et al. [29] studied the association between the use of *Helicobacter pylori* eradication treatment containing clarithromycin and cardiovascular outcomes. The exposure statuses of clarithromycin were compared during the case and control periods with time windows of 14 days. Each case acts as self-control and thus implicitly controls for time-invariant confounders such as the severity of underlying diseases. The OR estimated was 2.20 (95 % CI 1.23–3.95) which was in line with the conclusion of the SCCS analysis [IRR 3.38 (95 % CI 1.89–6.04)].

Risselada et al. [30] additionally applied CTC to address the issue of exposure-time trend bias in CCO. This study investigated whether the use of platelet aggregation inhibitors (PAI) and vitamin K antagonist (VKA) were associated with subarachnoid haemorrhage (SAH). Increased prevalence of PAI and VKA use were observed over the study period. For each case, the 1-month period preceding the index date was compared to the prior 11 control periods, which also had a length of 1 month each. The CCO analyses showed that SAH was positively associated with VKA use (OR 2.90; 95 % CI 1.27–6.65). However, after adjusting for the exposure-time trend in the CTC analyses, the effect decreased to non-significant levels (OR 1.98; 95 % CI 0.82–4.76).

## Other techniques

This article has so far focused on the pharmacoepidemiological techniques in drug safety hypothesis testing using automated databases. In recent years, there has been significant methodological development in using spontaneous reporting databases [31, 32] or prescribing data alone [33]. Methodologies such as disproportionality analysis [34] and prescription sequence symmetry analysis are increasingly popular [35]. However, due to the limitations of the quality and quantity of available data, these techniques are used mainly for drug safety hypothesis generation. Databases are also commonly used to perform descriptive drug utilisation studies. Drug utilisation studies are particularly useful in generating new information [36, 37] to decide whether further analytical studies are required [38].

Meta-analysis of observational studies (secondary analysis of data from existing observational studies) has also been increasingly applied for drug safety hypothesis testing [39–41]. The basic principles are the same as traditional meta-analysis of clinical trials, however, quality assessment is more challenging and the methodology is still evolving.

Finally, primary data collection in clinical settings is rarely done in developed countries in recent years. It is very labour-intensive and unlikely to be cost-effective in pharmacoepidemiology research. However, in some circumstances, it is still the only appropriate method for pharmacoepidemiology research such as monitoring drug administration errors in nursing staff [42, 43]. Special attention is needed to prevent interference in health professionals’ clinical responsibilities and patient care. Furthermore, appropriate training for researchers is crucial to ensure the validity and reliability of data collection. Consequently, primary data collection in clinical settings are being replaced gradually by automated databases in pharmacoepidemiology research in developed countries.

Table 1 summarises the strengths and limitations of various pharmacoepidemiological designs. Table 1 is intended to assist readers with selecting the appropriate design for future studies.

**Table 1:** Summary of the strengths and limitations of various pharmacoepidemiological designs

Method	Strengths	Limitations
Cohort	Exposure precedes outcomes Can explore multiple outcomes Allow rare exposures Can estimate the incidence of outcomes	Time- and resource- consuming Difficult to study rare outcomes
Case–control	Can explore multiple exposures Allow rare outcomes Quicker and cheaper (compared to cohort studies)	Difficult to study rare exposures Difficult to select proper cases and controls Cannot estimate the incidence of outcomes
Self-controlled case series (SCCS)	Eliminates time-invariant confounders Less data-intensive (compared to cohort or case–control studies) Temporal variables such as age can be accounted for by subdividing the observation period	Sensitive to time-variant confounders Cannot estimate the incidence of outcomes Not suitable when any of the following assumptions is violated Outcome events are rare or independent of each other Occurrence of outcome event is independent of the exposures Occurrence of outcome event or any subsequent conditions stemming from the event should not censor the observation period
Case-crossover (CCO)	Eliminates time-invariant confounders Less data-intensive (compared to cohort or case–control studies) Exposure-trend bias can be addressed by case–time–control (CTC) or case–case–time–control (CCTC)	Sensitive to time-variant confounders Cannot estimate the incidence of outcomes Not suitable when any of the following assumptions is violated Transient exposures and acute outcomes Exposure has a stable trend over time CTC and CCTC may reintroduce control-selection bias if the external controls are not well-matched

## Conclusion

Observational studies are essential to inform the safe use of medications. Classical epidemiological techniques such as cohort and case–control design have been widely used to investigate the association between drug exposure and clinical outcomes. Derived from cohort or case–control methods, case-only designs have been developed to eliminate time-invariable effect by self-matching. Such methods are gaining popularity among researchers in epidemiological and drug safety research. Finally, large databases provide useful platforms for observational studies to assess outcomes, including rare and long-term adverse events of medications.

## References

[1] Chan EW, Liu KQ, Chui CS, Sing CW, Wong LY, Wong IC (2014). Adverse drug reactions—examples of detection of rare events using databases. Br J Clin Pharmacol.

[2] Lai EC, Man KK, Chaiyakunapruk N, Cheng CL, Chien HC, Chui CS (2015). Databases in the Asia-Pacific region: the potential for a distributed network approach. Epidemiology.

[3] Farrington CP (1995). Relative incidence estimation from case series for vaccine safety evaluation. Biometrics.

[4] Maclure M (1991). The case-crossover design: a method for studying transient effects on the risk of acute events. Am J Epidemiol.

[5] Strom BL. Chapter 2: study designs available for pharmacoepidemiology studies. In: *Pharmacoepidemiology*. Wiley; 2006.

[6] Wong IC, Mawer GE, Sander JW (2001). Adverse event monitoring in lamotrigine patients: a pharmacoepidemiologic study in the United Kingdom. Epilepsia.

[7] He Y, Chan EW, Man KK, Lau WC, Leung WK, Ho LM (2014). Dosage effects of histamine-2 receptor antagonist on the primary prophylaxis of non-steroidal anti-inflammatory drug (NSAID)-associated peptic ulcers: a retrospective cohort study. Drug Saf.

[8] Chan EW, Lau WC, Leung WK, Mok MT, He Y, Tong TS (2015). Prevention of dabigatran-related gastrointestinal bleeding with gastroprotective agents: a population-based study. Gastroenterology.

[9] Wacholder S, McLaughlin JK, Silverman DT, Mandel JS (1992). Selection of controls in case–control studies. I. Principles. Am J Epidemiol.

[10] Ernster VL (1994). Nested case–control studies. Prev Med.

[11] Vinogradova Y, Coupland C, Hippisley-Cox J (2015). Use of combined oral contraceptives and risk of venous thromboembolism: nested case–control studies using the QResearch and CPRD databases. BMJ.

[12] Nordmann S, Biard L, Ravaud P, Esposito-Farese M, Tubach F (2012). Case-only designs in pharmacoepidemiology: a systematic review. PLoS One.

[13] Wang S, Linkletter C, Maclure M, Dore D, Mor V, Buka S (2011). Future cases as present controls to adjust for exposure trend bias in case-only studies. Epidemiology.

[14] Whitaker HJ, Farrington CP, Spiessens B, Musonda P (2006). Tutorial in biostatistics: the self-controlled case series method. Stat Med.

[15] Chui CS, Man KK, Cheng CL, Chan EW, Lau WC, Cheng VC (2014). An investigation of the potential association between retinal detachment and oral fluoroquinolones: a self-controlled case series study. J Antimicrob Chemother.

[16] Brauer R, Smeeth L, Anaya-Izquierdo K, Timmis A, Denaxas SC, Farrington CP (2015). Antipsychotic drugs and risks of myocardial infarction: a self-controlled case series study. Eur Heart J.

[17] Douglas IJ, Langham J, Bhaskaran K, Brauer R, Smeeth L (2013). Orlistat and the risk of acute liver injury: self controlled case series study in UK Clinical Practice Research Datalink. BMJ.

[18] Pratt NL, Roughead EE, Ramsay E, Salter A, Ryan P (2010). Risk of hospitalization for stroke associated with antipsychotic use in the elderly: a self-controlled case series. Drugs Aging.

[19] Weldeselassie YG, Whitaker HJ, Farrington CP (2011). Use of the self-controlled case-series method in vaccine safety studies: review and recommendations for best practice. Epidemiol Infect.

[20] Farrington CP, Anaya K, Whitaker H, Hocine MN, Douglas IJ, Smeeth L (2010). Self-controlled case series analysis with event-dependent observation periods. J Am Stat Assoc.

[21] Farrington CP, Whitaker HJ, Hocine MN (2009). Case series analysis for censored, perturbed, or curtailed post-event exposures. Biostatistics.

[22] Kuhnert R, Hecker H, Poethko-Muller C, Schlaud M, Vennemann M, Whitaker HJ (2011). A modified self-controlled case series method to examine association between multidose vaccinations and death. Stat Med.

[23] Man KK, Chan EW, Coghill D, Douglas I, Ip P, Leung LP (2015). Methylphenidate and the risk of trauma. Pediatrics.

[24] Maclure M, Mittleman MA (2000). Should we use a case-crossover design?. Annu Rev Publ Health.

[25] Suissa S (1995). The case–time–control design. Epidemiology.

[26] Suissa S (1998). The case–time–control design: further assumptions and conditions. Epidemiology.

[27] Horwitz RI, Feinstein AR (1980). The problem of “protopathic bias” in case–control studies. Am J Med.

[28] Greenland S (1996). Confounding and exposure trends in case-crossover and case–time–control designs. Epidemiology.

[29] Wong AY, Root A, Douglas IJ, Chui CS, Chan EW, Ghebremichael-Weldeselassie Y (2016). Cardiovascular outcomes associated with use of clarithromycin: population based study. BMJ.

[30] Risselada R, Straatman H, van Kooten F, Dippel DWJ, van der Lugt A, Niessen WJ (2011). Platelet aggregation inhibitors, vitamin K antagonists and risk of subarachnoid hemorrhage. J Thromb Haemost.

[31] de Bie S, Ferrajolo C, Straus SM, Verhamme KM, Bonhoeffer J, Wong IC (2015). Pediatric drug safety surveillance in FDA-AERS: a description of adverse events from GRiP Project. PLoS One.

[32] Osokogu OU, Fregonese F, Ferrajolo C, Verhamme K, de Bie S, Catapano M (2015). Pediatric drug safety signal detection: a new drug-event reference set for performance testing of data-mining methods and systems. Drug Saf.

[33] Pratt N, Chan EW, Choi NK, Kimura M, Kimura T, Kubota K (2015). Prescription sequence symmetry analysis: assessing risk, temporality, and consistency for adverse drug reactions across datasets in five countries. Pharmacoepidemiol Drug Saf.

[34] Star K, Iessa N, Almandil NB, Wilton L, Curran S, Edwards IR (2012). Rhabdomyolysis reported for children and adolescents treated with antipsychotic medicines: a case series analysis. J Child Adolesc Psychopharmacol.

[35] Roughead EE, Chan EW, Choi NK, Kimura M, Kimura T, Kubota K (2015). Variation in association between thiazolidinediones and heart failure across ethnic groups: retrospective analysis of large healthcare claims databases in six countries. Drug Saf.

[36] Ackers R, Murray ML, Besag FM, Wong IC (2007). Prioritizing children’s medicines for research: a pharmaco-epidemiological study of antiepileptic drugs. Br J Clin Pharmacol.

[37] Thompson PL, Spyridis N, Sharland M, Gilbert RE, Saxena S, Long PF (2009). Changes in clinical indications for community antibiotic prescribing for children in the UK from 1996 to 2006: will the new NICE prescribing guidance on upper respiratory tract infections just be ignored?. Arch Dis Child.

[38] Ackers R, Besag FM, Hughes E, Squier W, Murray ML, Wong IC (2011). Mortality rates and causes of death in children with epilepsy prescribed antiepileptic drugs: a retrospective cohort study using the UK General Practice Research Database. Drug Saf.

[39] Chui CS, Wong IC, Wong LY, Chan EW (2015). Association between oral fluoroquinolone use and the development of retinal detachment: a systematic review and meta-analysis of observational studies. J Antimicrob Chemother.

[40] He Y, Chan EW, Leung WK, Anand S, Wong IC (2015). Systematic review with meta-analysis: the association between the use of calcium channel blockers and gastrointestinal bleeding. Aliment Pharmacol Ther.

[41] Man KK, Tong HH, Wong LY, Chan EW, Simonoff E, Wong IC (2015). Exposure to selective serotonin reuptake inhibitors during pregnancy and risk of autism spectrum disorder in children: a systematic review and meta-analysis of observational studies. Neurosci Biobehav Rev.

[42] Bruce J, Wong I (2001). Parenteral drug administration errors by nursing staff on an acute medical admissions ward during day duty. Drug Saf.

[43] Jani YH, Ghaleb MA, Marks SD, Cope J, Barber N, Wong IC (2008). Electronic prescribing reduced prescribing errors in a pediatric renal outpatient clinic. J Pediatr.

